# Phytochemical Screening, Antioxidant and Antifungal Activities of *Aconitum chasmanthum* Stapf ex Holmes Wild Rhizome Extracts

**DOI:** 10.3390/antiox11061052

**Published:** 2022-05-26

**Authors:** Shah Rafiq, Nasir Aziz Wagay, Hosam O. Elansary, Mansoor Ahmad Malik, Irshad Ahmad Bhat, Zahoor Ahmad Kaloo, Abdul Hadi, Abed Alataway, Ahmed Z. Dewidar, Ahmed M. El-Sabrout, Kowiyou Yessoufou, Eman A. Mahmoud

**Affiliations:** 1 Plant Tissue Culture and Research Laboratory, Department of Botany, University of Kashmir, Srinagar 190006, India; shahrafiq.scholar@kashmiruniversity.net (S.R.); bhatirshad.scholar@kashmiruniversity.net (I.A.B.); zkaloo@uok.edu.in (Z.A.K.); 2 Department of Botany, Government Degree College Baramulla (Boys), Baramulla 193101, India; nasir.wagay1989@rediffmail.com; 3 Plant Production Department, College of Food & Agriculture Sciences, King Saud University, Riyadh 11451, Saudi Arabia; 4 Floriculture, Ornamental Horticulture, and Garden Design Department, Faculty of Agriculture (El-Shatby), Alexandria University, Alexandria 21545, Egypt; 5 Department of Geography, Environmental Management, and Energy Studies, University of Johannesburg, APK Campus, Johannesburg 2006, South Africa; kowiyouy@uj.ac.za; 6 Plant Pathology Laboratory, Department of Botany, University of Kashmir, Hazratbal, Srinagar 190006, India; mansoormalik.scholar@kashmiruniversity.net; 7 Prince Sultan Bin Abdulaziz International Prize for Water Chair, Prince Sultan Institute for Environmental, Water and Desert Research, King Saud University, Riyadh 11451, Saudi Arabia; aalataway@ksu.edu.sa (A.A.); adewidar@ksu.edu.sa (A.Z.D.); 8 Agricultural Engineering Department, College of Food and Agriculture Sciences, King Saud University, Riyadh 11451, Saudi Arabia; 9 Department of Applied Entomology and Zoology, Faculty of Agriculture (EL-Shatby), Alexandria University, Alexandria 21545, Egypt; elsabroutahmed@alexu.edu.eg; 10 Department of Food Industries, Faculty of Agriculture, Damietta University, Damietta 34511, Egypt; emanmail2005@yahoo.com

**Keywords:** antioxidant assays, IC_50_ value, phytochemical analysis, antifungal activity, extraction, MIC value

## Abstract

*Aconitum chasmanthum* Stapf ex Holmes, an essential and critically endangered medicinal plant from Kashmir Himalayas, was studied for its antioxidant and antifungal properties. The shade-dried powdered rhizome was extracted sequentially with hexane, ethyl acetate, and methanol. These subsequent fractions were evaluated for total phenolic content (TPC); total flavonoid content (TFC); antioxidant assays, such as 1,1-diphenyl 1-2-picryl-hydrazyl (DPPH); ferric-reducing antioxidant power (FRAP); superoxide radical scavenging (SOR); hydroxyl radical scavenging (OH) and antifungal activity using the poisoned food technique. Highest TPC (5.26 ± 0.01 mg/g) and TFC (2.92 ± 0.04 mg/g) were reported from methanolic extracts. The highest values of radical scavenging activities were also observed in methanolic extracts with IC_50_ values of 163.71 ± 2.69 μg/mL in DPPH, 173.69 ± 4.91 μg/mL in SOR and 159.64 ± 2.43 μg/mL in OH. The chemical profile of ethyl acetate extract was tested using HR-LCMS. Methanolic extracts also showed a promising inhibition against *Aspergillus niger* (66.18 ± 1.03), *Aspergillus flavus* (78.91 ± 1.19) and *Penicillium notatum* (83.14 ± 0.97) at a 15% culture filtrate concentration with minimum inhibitory concentration (MIC) values of 230 μg/mL, 200 μg/mL and 190 μg/mL, respectively. Overall, the methanolic fractions showed significant biological potential, and its pure isolates might be used to construct a potential new medicinal source.

## 1. Introduction

From the dawn of human history, plants and their products have been employed as natural medicines, nutritional supplements and medications [1]. Traditional medicine is still utilised by 60–80 percent of the world’s population to treat common ailments [2]. The major reasons for utilizing folk medicine as a medical service source are accessibility, affordability and cultural beliefs [3,4]. Because of their medicinal and nutritional properties, as well as their large supply of phytochemical compounds, plants have been shown to be absolutely crucial. Plant species, variety, extraction and/or processing procedures, and the growing environment all influence the efficacy of natural antioxidants derived from plants.

Total phenolics, antioxidant activity, anticancer activity and enzyme inhibition of Indian medicinal and aromatic plant extracts or pure compounds isolated from them are being tested by different methods. The comparison of the pharmacological activity of phytochemical components isolated from plants is becoming increasingly popular [5]. Because of their advantageous pharmacological action, economic feasibility and low toxicity, plant medicinal capabilities have been examined in the wake of contemporary systematic breakthroughs throughout the world [6]. Flavonoids, phenolics, sterols, alkaloids, carotenoids and glucosinolates are only a few of the antioxidant-containing bioactive compounds found in plants [7].

*Aconitum chasmanthum* Stapf ex Holmes (Ranunculaceae), a critically endangered and well-known medicinal plant, is widely utilised in traditional and folk remedies across Southeast Asia [8]. Raw rhizomes are harvested from high-altitude alpine and subalpine meadows in the Western Himalayas. After being mitigated, rhizomes are used in several Ayurvedic formulations and homoeopathic systems of medicine, and have been sold under the name ‘Vatsanabha’ since ancient times. Its rhizomes have antifungal, insecticidal and antibacterial properties [9], and have been used to treat pain and inflammation, as an appetizer [10], as an antirheumatic [11], as an ointment for the treatment of abscesses and boils [12], to treat heart disease [13,14], fever, coughs, asthma and snake bites [14]; and as a antidiarrhoeal, anodyne, anti-inflammatory, antidiabetic, neurasthenic, astringent, and to treat tonsillitis and colds [15,16].

An antioxidant is a chemical that slows down or stops the oxidation of a substrate at low doses. Antioxidant chemicals act by a number of chemical mechanisms, such as hydrogen atom transfer (HAT), single electron transfer (SET) and transition metal chelation [17,18]. Antioxidants serve a physiological role by protecting cell structures from damage caused by free radicals in chemical reactions. According to an increasing amount of data, free radicals play a critical role in many essential physiological activities and oxidative stress may play a part in the etiology of prevalent illnesses, including atherosclerosis, chronic renal failure and diabetes mellitus

A billion individuals, more or less, have grave fungal diseases of the skin, nails and hair; nearly a billion people have significant fungal illnesses that affect their lives or are lethal; and another billion have severe fungal infections that have a significant impact on their lives or are deadly [19]. The gravity of infections varies, ranging from symptomless infections of the skin to fatal systemic infections. Furthermore, the death rate related to fungal illness is >1.6 million, which is comparable to tuberculosis and >3 times higher than malaria [20]. The key factors of differences in occurrence and frequency of fungal illness throughout the globe include socioeconomic, geo-ecological and the growing figure of at-risk inhabitants.

Aspergilli have always been a part of humans’ surroundings, but it was not until the middle of the nineteenth century that they were identified as active participants in decay processes, sources of animal and human illness or fermenting organisms able to produce valuable primary metabolites [21]. Infections with *Aspergillus* spp. result in high sickness and mortality [22]. *Aspergillus fumigatus, Aspergillus terreus* and *Aspergillus flavus* are the most common sources of infection. *A. niger* is less frequently connected with invasive illness [22]. *Otomycosis* [23], dermatological and respiratory illness [24] have all been linked to *A. niger.* There have been few reports of *A. niger* pneumonia. Infections caused by *A. niger* are uncommon in people with hematological disorders but are the most prevalent cause of otomycosis in immunocompetent individuals [25,26]. According to recent figures, 30 lakh cases of chronic respiratory infections, 2.23 lakh cases of cryptococcal meningitis complicating HIV/AIDS, 70,000 cases of invasive candidiasis, 2.5 lakh cases of invasive aspergillosis, 5 lakh cases of *Pneumocystis jirovecii* pneumonia, 1 lakh cases of disseminated histoplasmosis and more than 1 billion cases of fungal asthma occur each year [19,27,28].

*A. flavus* is a kind of mycotoxigenic fungus capable of producing B aflatoxins. *Second only to A. fumigatus, A. flavus is the leading source of human invasive aspergillosis.* It is also the most common *Aspergillus* species to infect insects [29], and it may cause illnesses in economically significant crops, such as maize and peanuts, as well as create strong mycotoxins. *A. flavus* is a saprophyte that lives in soils all over the world and causes illnesses on a variety of major agricultural crops, including maize (spike rot), peanuts (yellow mold) and cottonseed during the harvest [15,30]. Both animals and humans are infected by the fungus, either through tainted feed (aflatoxicosis or pancreatic cancer) or invasive growth (aspergillosis), which is frequently deadly in vulnerable people [16]. A broad variety of plant products, including fruits, such as grapes, degrade and decompose rapidly after harvest due to *Penicillium* spp. [31,32]. A broad range of fruits, including grapes, are susceptible to assault by these fungi, which produce mycotoxins, particularly when they are stored. A wide variety of toxic mycotoxins and carcinogenic chemicals, such as citrinin and patulin (as well as others), are generated by *Penicillium* species [33,34].

Conventional pesticides and fungicides used in agriculture have been linked to a number of environmental and human health issues [35]; resistant strains of plant diseases may be developed or already exist due to widespread usage of chemical treatments. Pesticide residues may be discovered in the product of organically grown plants; thus, producers have a moral obligation to minimize the use of pesticides. A new approach to controlling and curing plant diseases is therefore required [36]. To compete with current strategies against plant diseases and pests, these approaches must be effective, safe for the environment and people and economically profitable.

As a result of this research, in the last several decades, the antimicrobial and antifungal properties of various extracts and their ingredients, such as essential oils, have been studied and attention is focused on the use of these natural materials in alternative plant-protection measures [37].

The purpose of this study is to look into the phytochemical profile, antioxidant and antifungal properties of *Aconitum chasmanthum*, a threatened species endemic to the Kashmir Himalayan region.

## 2. Materials and Methods

### 2.1. Collection of Plant Material

In September 2020 (flowering season), the whole species (5 plants) of *A. chasmanthum* was obtained from Razdhan pass (Bandipora), Jammu and Kashmir, India (34°33′46.7″ E and 74°37′76.7″ N, 3423 m asl). The specimen was identified and validated at the Centre of Biodiversity and Taxonomy, Department of Botany, University of Kashmir. A herbarium specimen was deposited at Kashmir University Herbarium under Voucher Specimen No. 2939-(KASH).

### 2.2. Chemicals

For phytochemical extraction, hexane, ethyl acetate and methanol were utilized as solvents. All of the solvents were purchased from Sigma Aldrich, Pvt. Ltd. in Mumbai, India. Folin–Ciocalteu’s phenol reagent (2N), sodium carbonate (Na_2_CO_3_), 2,4,6-tripyridyl-s-triazine (TPTZ), Phenazine methosulphate (PMS) were all purchased Sigma Aldrich, Pvt. Ltd. in Mumbai, India. Gallic acid, aluminum chloride (AlCl_3_), rutin, DPPH, sodium acetate (CH_3_COONa), Tris-HCl, nicotinamide adenine dinucleotide (NADH), nitro blue tetrazolium (NBT), salicylic acid, ethanol, ferrous sulphate (FeSO_4_), hydrogen peroxide (H_2_O_2_), Sabouraud’s dextrose broth (SDB) and potato dextrose agar (PDA) were purchased from Hi-Media, India.

### 2.3. Cold Extraction of Wild Rhizomes of A. chasmanthum

Considering its critically endangered status, wild rhizomes (5 g), stem, leaves and flowers were cleaned and cut into tiny pieces, then shade-dried at ambient temperature before being processed into powder with a mechanical grinder. The rhizome powder was then sequentially extracted using a series of solvents, including hexane, ethyl acetate and methanol, over a period of 48 h, with occasional shaking and using intermittent heating over a water bath at their respective boiling temperatures. Whatman filter paper No. 1 was used to filter the extracts. The filtrate was collected and concentrated in a water bath, with the leftovers being discarded. The dried extracts were labeled and kept in glass vials at 4 °C for subsequent testing.

### 2.4. Qualitative Phytochemical Analysis

The subsequent fractions obtained with hexane, ethyl acetate and methanol were qualitatively analyzed for secondary metabolites, such as phenolics, alkaloids, glycosides, tannins, flavonoids, terpenes, saponins and steroids, using conventional procedures [38,39,40,41].

### 2.5. Identification of Bioactive Molecules by Liquid Chromatography Coupled with High-Resolution Mass Spectrometry

The rhizome of *A. chasmanthum* is, medicinally, an important part of the plant, a rich source of diterpenoid alkaloids and the only part of the plant used in traditional medicines. Therefore, High-Resolution Liquid Chromatography and Mass Spectrometry (HR-LCMS) analysis, antifungal and antioxidant analyses of the rhizome extract only were conducted. HR-LCMS analysis was performed on the ethyl acetate extract. A Sophisticated Analytical Instrument Facility (SAIF), IIT Bombay, Powai, Mumbai, India, was used for the HR-LCMS of the samples. A chemical fingerprint of the plant extract was prepared by high-resolution liquid chromatography and mass spectrometry model-1290 Infinity ultra-high performance liquid chromatography (UHPLC) System, 1260 infinity Nano HPLC with Chipcube, 6550 iFunnel Q-TOFs (Agilent technologies, Santa Clara, CA, USA), having specification of direct infusion mass analysis (MS, MS/MS) with ESI positive mode and negative mode ionizations. The mass range of 50 to 3200 amu was specified for the acquisition procedure, having a mass accuracy of less than 1 ppm, with a scanning rate of each spectrum per second. The analysis was performed in ESI positive and negativemode ionizations.

### 2.6. Total Phenolic Content (TPC)

The TPC of various extracts of wild rhizomes was assessed by using a modified spectrophotometric approach of Singleton et al. [42]. The phenolic composition was identified by the change in color of the Folin–Ciocalteu reagent from yellow to blue. Folin’s reagent (2N) was incubated with plant extract (1 mg/mL) in the presence of 4 mL of 20% Na_2_CO_3_. The mixture was kept at an ambient temperature for 20 min. Light absorbance of samples was measured at 765 nm. The samples were prepared in triplicate and the mean value absorbance was determined. The gallic acid standard curve was used to determine the amount of phenols in the extracts, and the results were expressed in mg gallic acid equivalent (GAE) per gram of plant extract from the calibration curve, y = 0.0917x + 0.0631, R^2^ = 0.9978.

### 2.7. Total Flavonoid Content (TFC)

A spectrophotometric technique with specified modifications was used to determine the flavonoids concentrations (Quitter et al. [43]). Each sample contained 1 mL of different extracts (hexane, ethyl acetate and methanol) in a concentration of 1 mg/mL of 2% AlCl_3_ in respective solvents. The samples were kept at normal room temperature for 1 h. Then, the samples’ absorbance was obtained at 415 nm. As a control, methanol was employed. Triplicates of each sample were prepared, then the mean of the absorbance for each sample was determined. The amount of flavonoids in extracts was calculated using the rutin equivalent (mg of RU/g of extract) obtained form the calibration curve, y = 0.1664x + 0.0436, R^2^ = 0.9988.

### 2.8. Biological Activity

#### 2.8.1. Antioxidant Assays

Different antioxidant assays were used in this work to analyze extracts of wild *A. chasmanthum* rhizomes for free radical scavenging activity.

##### DPPH Radical Scavenging Assay

With minor changes, the technique of Braca et al. [44] was used to investigate the DPPH radical scavenging activity of plant extracts. Different amounts of wild rhizome extract (50–250 μg/mL) were diluted with 1 mL of 0.5 mM DPPH solution. The reaction mixture was stirred often before being kept in the dark at room temperature for 30 minutes. After incubation, the sample’s absorbance was determined spectrophotometrically (Shimadzu 1900i, Kyoto, Japan) against methanol at 517 nm (used as a blank). The increase in DPPH free radical scavenging capacity was indicated by the reduction in absorbance. The percentage of DPPH free radical inhibition was calculated using the following formula:

$$\%\;\mathrm{inhibition}=\frac{\left(A_{C}-A_{S}\right)}{A_{C}}\times 100$$

where *A_C_* signifies the absorbance of the control (without the plant extract), and *As* denotes the sample’s absorbance (reaction mixture containing plant extracts). As a control, α-tocopherol was used. The test was repeated thrice.

##### Ferric-Reducing Antioxidant Power (FRAP)

With minor adjustments, the FRAP test was carried out in accordance with the technique provided by Pang et al. [45]. In a 10:1:1 ratio, 300 mM of sodium acetate buffer solution at pH 3.6, 20 mM of ferric chloride (FeCl_3_) solution and 10 mM of TPTZ (2,4,6-tripyridyl-s-triazine) solution were prepared and combined. Before use, the FRAP reagent was pre-heated to 37 °C and the plant extracts were combined with a 1.9 mL FRAP reagent. The absorbance of each sample was determined at 593 nm after a 10 min incubation period at room temeperature. The FRAP values were computed and represented as μM of ferrous equivalent Fe (II) per g of sample based on the dry weight of the samples. The test was repeated thrice.

##### Superoxide Anion Radical Scavenging Activity (SOR)

The SOR was performed with slight modifications utilizing the Liu and Ng [46] approach. The radicals were produced in a 16 mM Tris-HCl buffer at pH 8 containing 10 mM phenanzine methosulphate (PMS), 78 mM nicotinamide adenine dinucleotide (NADH), 50 mM nitroblue tetrazolium (NBT) and rhizome extracts at concentrations of 50 μL, 100 μL, 150 μL, 200 μL and 250 μL. To examine the reactions between NBT and SOR radicals, the production of purple formazan color was measured spectrophotometrically at 560 nm. The addition of wild rhizome extract to the reaction mixture, on the other hand, inhibits NBT reduction by quenching superoxide radicals (O^2−^_)_. The reaction mixture’s decreased absorbance showed that it could scavenge more superoxide anion. The formula below was used to compute the percent inhibition of SOR.

$$\%\;\mathrm{inhibition}=\frac{\left(A_{C}-A_{S}\right)}{A_{C}}\times 100$$

where *A_C_* is blank absorbance, and *As* is sample absorbance. BHT was employed as a positive control. The test was repeated thrice.

##### Hydroxyl Radical Scavenging (OH^−^)

The salicylate technique developed by Zhao et al. [47] was utilized to assess the hydroxyl radical scavenging capacity with minor changes. A total of 1 mL plant extracts at concentrations of 50 μL, 100 μL, 150 μL, 200 μL, and 250 μL were added to a 4 mL reaction mixture containing 1 mL of salicylic acid dissolved in 100% ethanol (9 mM), FeSO_4_ (6 mM) and H_2_O_2_ (24 mM). H_2_O_2_ was added to the mixture and incubated for 30 minutes at room temperature to initiate the reaction. The absorbance was measured at 510 nm.

The OH radical scavenging percentage was calculated using the next equation:

$$\%\;\mathrm{inhibition}=\frac{\left(A_{C}-A_{S}\right)}{A_{C}}\times 100$$

where *A_C_* represents the absorbance of a blank (without plant extract) and *As* represents the absorbance of the sample. The test was repeated thrice.

#### 2.8.2. Antifungal Activity

The antifungal effect of various dosages of *A. chasmanthum* rhizome extracts (ethyl acetate and methanol) against *Aspergillus flavus, Aspergillus niger* and *Penicillium notatum* was tested in the current study.

##### Test Microorganisms

*Aspergillus flavus*, *Aspergillus niger* and *Penicillium notatum* were the fungi used. The fungal strains were supplied by the Plant Pathology Laboratory (Department of Botany, University of Kashmir). All of the strains were maintained and cultivated on the Potato Dextrose Agar (PDA) medium.

##### Poisoned Food Method

Various extracts (ethyl acetate and methanol) of wild rhizomes of *A. chasmanthum* were investigated for their efficacy on the inhibition of mycelial growth of pathogenic fungus utilizing the food poisoning technique [48,49]. Extracts (1 mg/mL) were prepared by dissolving in 1% dimethyl sulfoxide (DMSO). Plant extract concentrations of 5%, 10% and 15% were obtained by adding an appropriate quantity of the corresponding solvent to a standard concentration (1 mg/mL). PDA medium with various concentrations of rhizome extracts was sterilized and put on labeled Petri plates. The above-mentioned plant extract concentrations were combined with PDA medium and then solidified on sterilized Petri plates in a laminar airflow setting. The Petri plates were infected after solidification by putting 5 mm mycelial discs of the specific fungus at the center of each plate. The discs were collected from colonies that were rapidly growing. Triplicates of each dosage were retained. At a temperature of 24 ± 2 °C, the Petri plates were inspected for mycelial development after seven days of incubation. As a control, PDA plates without rhizome extracts (1% DMSO) were utilized and hexaconazole (1 mg/mL) was considered as the positive control. The inhibition of growth (as percentage) caused by different treatments at various dosages was calculated using the below equation:

$$\%\;\mathrm{inhibition}=\frac{\left(C-T\right)}{C}\times 100$$

where *C* signifies the fungal colony’s average diameter (mm) in the control and *T* denotes the fungal colony’s average diameter (mm) in the test.

##### MIC (Minimum Inhibitory Concentrations) of the Plant Extracts

An antifungal drug’s MIC is expressed as the lowest dose showing no growth is visible in the wells (80–100% inhibition) when examined visually. A micro-broth dilution procedure, as reported by Weigand et al. [50] and in accordance with the Clinical and Laboratory Standards Institute (CLSI) methodology [51], was used to determine each extract’s MIC. Sabouraud’s dextrose broth (SDB) was inoculated with a fresh colony of fungal isolates and incubated at 37 °C for 4 h, equating to 2 × 10^5^ CFU (colony forming unit) adjusted to the 0.5 McFarland standard value. The plates were cultured at 37 °C for 24 to 48 hours before being checked for fungal growth to determine the MIC. After 48 h of incubation in the micro-broth dilution, the MIC values were obtained using the lowest concentration of extract with no fungal growth. Plant extracts ranging from 150 to 300 µg/mL were employed to assess the antifungal activity of all previously identified fungus species.

### 2.9. Statistical Analysis

For each experiment, the results are shown as mean ± SE (standard error). Each of the experiments listed and compared in each table was examined in parallel studies, with statistical analysis conducted separately for each test. For antioxidant testing, two-way analysis of variance (ANOVA) with Tukey’s test of multiple comparisons was performed using GraphPad prism 8. IC_50_ values were calculated using GraphPad prism 8 and MS Excel 2019 by analyzing their linear regression equation. Scatter plots demonstrating Pearson’s pairwise correlation matrix was prepared using R (version 3.2). Pearson correlation coefficient and heatmap between antioxidant activity and the samples’ phenolic and flavonoid levels were analyzed using GraphPad prism 8. Tukey’s one-way analysis of variance (ANOVA) was used to analyze the antifungal data (SPSS 23, SPSS Inc., Chicago, IL, USA). Statistical significance was defined at *p* ≤ 0.05.

## 3. Results

### 3.1. Qualitative Phytochemical Analysis

The phytochemical study of different parts of wild *A. chasmanthum* revealed a moderate to a significant presence of many bioactive components, such as phenolics, alkaloids, glycosides, tannins, flavonoids, terpenes, saponins and steroids, as indicated in Table 1.

### 3.2. Phytochemical Composition

*A. chasmanthum* ethyl acetate extract was analysed using the HR-LCMS method, and 35 compounds were identified. Table 2 lists all of the compounds that have been identified, together with their *m*/*z*, adduct, and precise mass, as well as the chemical class they belong. 

**Figure 1:**
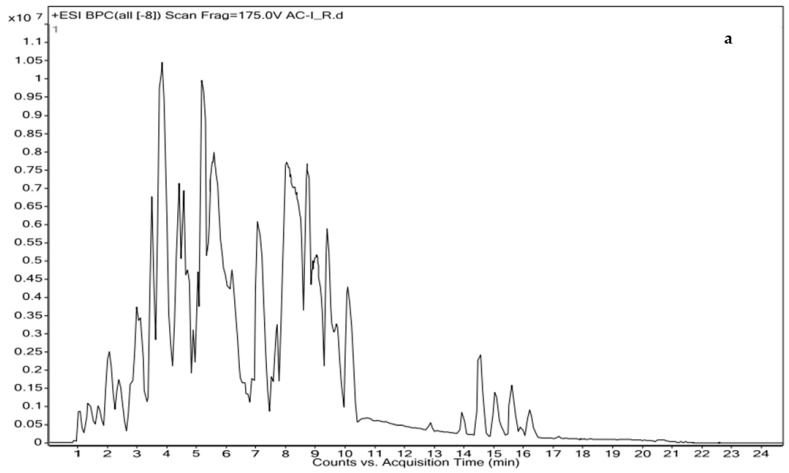

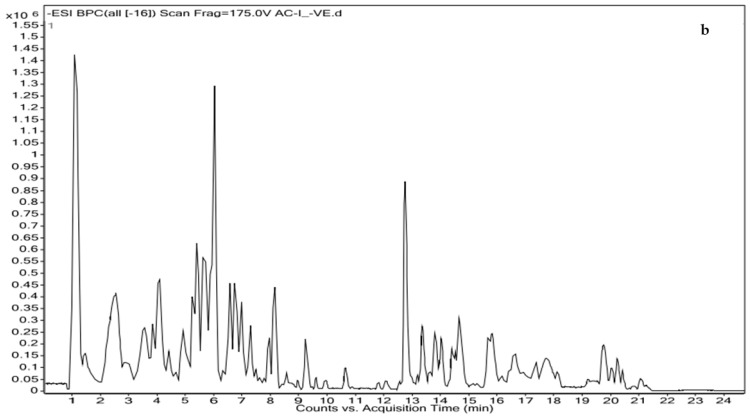
Main compounds identified in *A. chasmanthum* ethyl acetate extract of wild rhizome using the HR-LCMS technique. (**a**) ESI positive mode and (**b**) ESI negative mode.

Ethyl acetate extract of *A. chasmanthum* wild rhizome was dominated by alkaloids, especially diterpenoid alkaloids, flavonoid derivative, phenolic derivative, flavonoid O-glycosides, quinoline alkaloid, terpenes, coumaric acid derivatives, triterpenoids, terpene glycoside and carotenoids. The chromatograms (Figure 1a,b) respectively showed major peaks, indicating the presence of various bioactive compounds.

### 3.3. Total Phenolic Content (TPC)

The total phenolic content of the extracts of *A. chasmanthum* increased as follows: methanolic extract > ethyl acetate extracts > hexane extracts. Methanolic extracts had the maximum phenolic content in the wild rhizome, at 5.26 ± 0.01 mg GAE/g extract, whereas the phenolic values in ethyl acetate and hexane extracts were 1.12 ± 0.04 mg GAE/g extract and 0.42 ± 0.06 mg GAE/g extract, respectively.

### 3.4. Total Flavonoid Content (TFC)

TFC was measured as the mg/g of plant extract in rutin equivalents for various plant extracts. The TFC of several extracts of *A. chasmanthum* wild rhizomes improved as follows: methanolic extract > ethyl acetate extracts > hexane extracts. The highest flavonoid content was 2.92 ± 0.04 mg rutin/g extract in the methanolic extract, followed by 0.24 ± 0.02 mg rutin/g extract flavonoid in ethyl acetate and 0.11 ± 0.02 mg rutin/g extract flavonoid in hexane extract, respectively.

### 3.5. Antioxidant Assays

DPPH (2,2-diphenyl-1-picrylhydrazyl) radical scavenging, ferric-reducing power (FRP), superoxide anion radical scavenging and hydroxyl radical scavenging (OH^−^) were used to analyze extracts of wild *A. chasmanthum* rhizomes for free radical scavenging effects in this work. All of the approaches combined present a more accurate assessment of antioxidant capabilities, and the results show that inhibitory action was concentration-dependent.

#### 3.5.1. DPPH Radical Scavenging Activity

**Figure 2:**
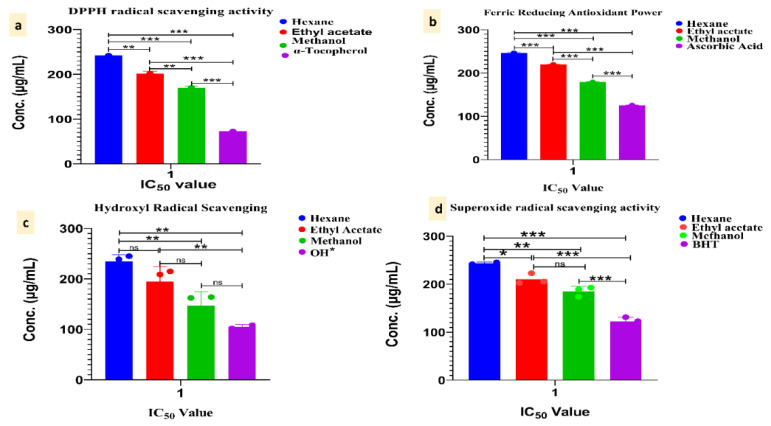
IC_50_ value along with Tukey’s multiple comparisons tests: (**a**) DPPH radical scavenging activity, (**b**) ferric-reducing antioxidant power, (**c**) hydroxyl radical scavenging activity and (**d**) superoxide radical scavenging activity. The significance level represented as stars (*** *p* < 0.0000, ** *p* < 0.001, * *p* < 0.01, ns = not significant).

The DPPH radical scavenging test revealed that various extracts had varying scavenging capabilities. The various extracts revealed solvent and concentration-dependent scavenging capacities, as per the results of this experiment. Methanolic extracts had a stronger scavenging action than other extracts, with the highest values of 65.58 ± 0.95%, followed by ethyl acetate extract with the highest value of 59.59 ± 0.75% and hexane with the highest inhibition of 49.99 ± 0.95% (Table 3). The IC_50_ values of hexane extract, ethyl acetate and methanol, respectively, were 263.01 ± 1.70 μg/mL, 199.50 ± 1.99 μg/mL and 163.71 ± 2.69 μg/mL (Figure 2a, Table 3).

#### 3.5.2. Ferric-Reducing Antioxidant Power (FRAP)

As shown in Table 3, the reduction power of *A. chasmanthum* wild rhizome extracts was concentration-dependent. The greater the sample’s reducing activity, the higher the absorbance value. With 50–250 μg/mL, the FRAP value at 593 nm increased from 7.72 ± 0.41 to 50.26 ± 0.48 μM Fe II/g DW for hexane extract, 11.67 ± 0.88 to 54.30 ± 0.75 μM Fe II/g DW for ethyl acetate extract and 16.31 ± 0.24 to 68.64 ± 0.37 μM Fe II/g DW for methanolic extract (Table 4). The IC_50_ values of hexane extract, ethyl acetate and methanol, respectively, were 246 ± 0.56 μg/mL, 219.71 ± 0.32 μg/mL and 179.11 ± 0.73 μg/mL (Figure 2b, Table 4).

#### 3.5.3. Superoxide Anion Radical Scavenging Activity (SOR)

The methanolic extracts at 250 μg/mL demonstrated the highest percentage inhibition of 60.0 ± 0.21, followed by ethyl acetate 54.75 ± 1.07 and hexane 50.86 ± 0.75 (Table 5). The IC_50_ values of hexane, ethyl acetate and methanol rhizome extract, respectively, were 243.54 ± 0.34 μg/mL, 192.23 ± 0.39 μg/mL and 178.33 ± 0.91 μg/mL (Figure 2c, Table 5).

#### 3.5.4. Hydroxyl Radical Scavenging (OH^−^) Activity

The highest percentages of OH radical reduction was shown by methanolic extract (67.24 ± 0.49), followed by ethyl acetate (58.98 ± 0.70) and hexane extracts (51.86 ± 1.95). The IC_50_ values of hexane, ethyl acetate and methanol rhizome extract, respectively, were 238.85 ± 0.23 μg/mL, 208.85 ± 0.45 μg/mL and 159.64 ± 0.42 μg/mL (Figure 2d, Table 6).

#### 3.5.5. Relationship between Different Solvent Systems in Total Phenolic, Flavonoid and Antioxidant Activity

**Figure 3:**
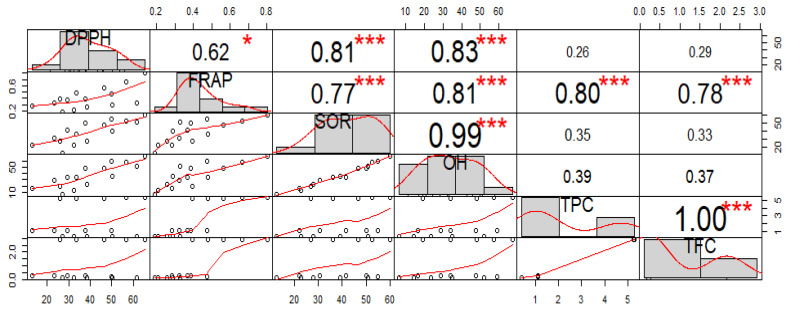
Scatter plots demonstrating Pearson’s pairwise correlation matrix below and along the diagonal in the plot for six variables: DPPH, FRAP, SOR, OH, TPC and TFC. Above the diagonal are the values of the correlation plus the significance level represented as stars (*** *p* < 0.0000, * *p* < 0.01).

**Figure 4:**
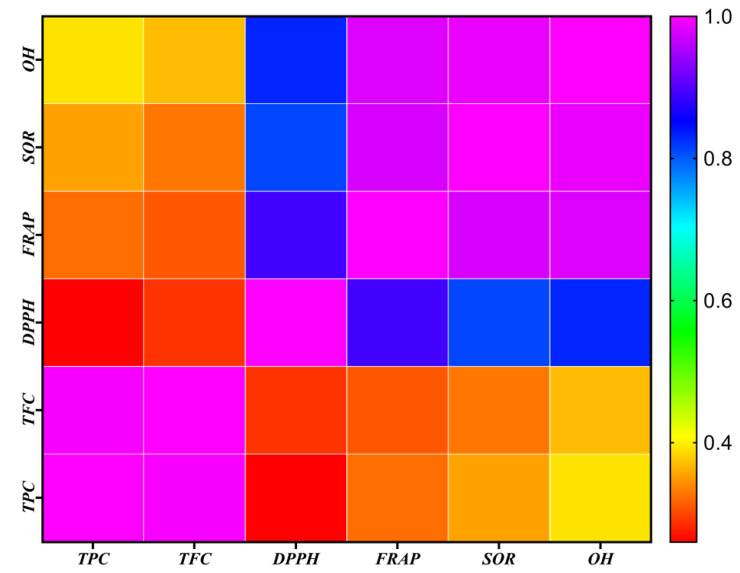
Pearson correlation coefficients for total phenolic content (TPC), total flavonoid content (TFC), and antioxidant activities 1-diphenyl 1-2-picryl-hydrazyl (DPPH), ferric-reducing antioxidant power (FRAP), superoxide radical scavenging (SOR) and hydroxyl radical scavenging (OH^−^) activity of *A. chasmanthum* extracts.

For measuring the associations between various extracts of *A. chasmanthum*, Pearson’s correlation coefficient was utilized. Comparative and correlative analyses were performed on the results of the various antioxidant tests used in the present investigation on the various A. chasmanthum extracts. Figure 3 depicts the relationship between the outcomes of many antioxidant tests. The DPPH radical scavenging activity showed a weak correlation with the TPC (*R*^2^ = 0.26) and TFC (*R*^2^ = 0.29). Antioxidant assays, such as FRAP (R^2^ = 0.80 ***), showed a strong correlation with TPC, while SOR (R^2^ = 0.35), OH (R^2^ = 0.39) and DPPH radical scavenging assay (R^2^ = 0.26) exhibited a week correlation with TPC (Figure 3). In addition, TFC showed a good correlation with FRAP (R^2^ = 0.78 ***), SOR (R^2^ = 0.33), OH (R^2^ = 0.37) and DPPH radical scavenging activity (R^2^ = 0.29). Figure 4 depicts the Pearson correlations and degrees of significance for the association between total phenolic content (TPC), total flavonoid content (TFC) and antioxidant (FRAP, DPPH, SOR, OH) activities in different extraction solvents.

### 3.6. Antifungal Activity

The technique of poisoned food was used to test the inhibitory activity of different doses of rhizome extracts of *A. chasmanthum* against *Aspergillus flavus, Aspergillus niger* and *Penicillium notatum*, and the result is presented in Table 7. The average diameter of test fungus colonies in poisoned food plates was much less than colony diameter in control plates, indicating that extracts had antifungal potential. The level of inhibition was proportional to the concentration of extracts. The sensitivity to extracts of the fungi examined demonstrated that increasing the concentration of plant extracts inhibited the test fungus’s mycelial growth.

**Figure 5:**
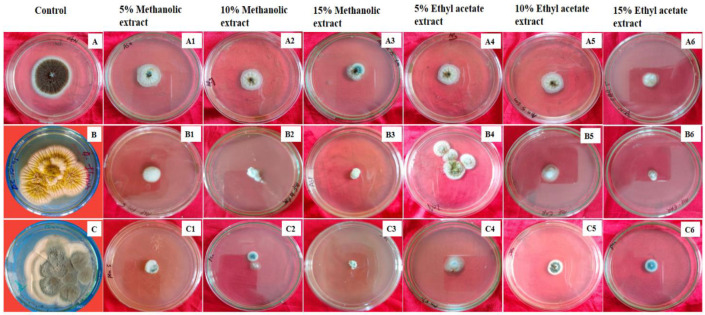
Inhibition of mycelia growth in *Aspergillus niger*, *Aspergillus flavus* and *Penicillium notatum* by methanolic extract (ME) and ethyl acetate extracts (EAEs) at 5%, 10% and 15% after five days of incubation. (**A**) Control *Aspergillus niger*, (**B**) control *Aspergillus flavus* and (**C**) control *Penicillium notatum*. (**A1**–**A3**) Inhibition of mycelia growth in *Aspergillus niger* by 5%, 10% and 15% methanolic extracts, respectively. (**B1**–**B3**) Inhibition of mycelia growth in *Aspergillus flavus* by 5%, 10% and 15% methanolic extracts, respectively. (**C1**–**C3**) Inhibition of mycelia growth in *Penicillium notatum* by 5%, 10% and 15% methanolic extracts, respectively. (**A4**–**A6**) Inhibition of mycelia growth in *Aspergillus niger* by 5%, 10% and 15% ethyl acetate extracts, respectively. (**B4**–**B6**) Inhibition of mycelia growth in *Aspergillus flavus* by 5%, 10% and 15% ethyl acetate extracts, respectively, and (**C4**–**C6**) inhibition of mycelia growth in *Penicillium notatum* by 5%, 10% and 15% ethyl acetate extracts, respectively.

The highest inhibition of mycelial development was found with a 15% concentration of methanolic extracts (MEs) against *Penicillium notatum*, resulting in a 83.14 ± 0.97% (Table 7, Figure 5C3) reduction in mycelial growth above control, while the minimum inhibition of mycelial growth was observed with 5% ethyl acetate extract (EAE) against *Aspergillus flavus* resulting in 36.75 ± 3.72 (Table 7, Figure 5B4) inhibition of mycelial growth above control.

## 4. Discussion

A plant’s therapeutic value is related to its abundance of phytoconstituents [52]. They contribute to human health in a variety of ways, including antioxidant properties, cell differentiation effects, improved detoxification enzyme activity, DNA metabolism effects, DNA repair maintenance, cancer cell death and cell proliferation reduction. Phytochemicals have been ingested by cultures all over the world from time immemorial, but scientific evidence to back this up is lacking. The study, development and marketing of functional bioactive components and nutraceuticals are gaining popularity across the world [53]. Consumer knowledge of the link between nutrition, health and illness has led to an increase in the intake of plant-derived bioactive components in the last two decades. Humans have always been on the lookout for natural goods that can boost biological functioning and help people live longer, better and fitter lives [54]. Plants are one of nature’s most valuable treasures, with a lengthy history of traditional use as food and medicine dating back to antiquity. The global growth in human health issues, on the other hand, has always posed a serious challenge to medical science [53].

Both extract of ethyl acetate and methanol of *A. chasmanthum* exhibited larger yields of phenolic components than hexane extract. This is owing to the polarity and eluent strength differences between hexane, ethyl acetate and methanol. For extracting compounds with a wide range of polarity, methanol is the most effective solvent [55]. According to studies on phenolic compounds’ biological activities, phenols serve a crucial role in antioxidant activity by quenching free radicals, singlet oxygen (O^2−^) or metal ions (Fe^2+^) due to their lower redox potential [56,57]. Various antioxidant investigations on plant extracts [58,59,60] have found a high link between a plant’s total phenolic content and its resulting antioxidant capabilities. Methanolic extracts had the greatest total flavonoid level, which was similar to what had been observed in phenolic compositions. Flavonoids have biological actions, such as free radical scavenging, metal chelating activity, cardio-protective and hepatoprotective, anti-inflammatory and anticancer activities [61,62]. Our results show that methanolic extracts have the highest radical scavenging effect, followed by ethyl acetate and hexane. The kind of extracting solvent utilized has an impact on the antioxidant activity of plant extracts. The type of extraction solvents has been shown to alter both the production of phytochemical ingredients and, as a result, their cumulative antioxidant activity. This is due to the vast range of chemical characteristics and polarity of phytoconstituents, which result in varied solvent solubilities [63,64].

Qualitative phytochemical analysis of the rhizome, stem, leaves and flower extracts revealed tannins, alkaloids, saponins, glycosides, flavonoids and steroids in the three extracts of hexane, ethyl acetate and methanol. HR-LCMS study of the ethyl acetate plant extract of the *A. chasmanthum* rhizome showed, respectively, 15 and 20 major peaks in ESI +ve and ESI −ve modes. When comparing the high-resolution liquid chromatograms and mass spectra of constituents with the main library, all these compounds were characterized and probably identified. The identified compounds were mostly diterpene alkaloids [65,66], flavonoid and terpene glycosides [67] and phenol derivatives. However, it is the first report for *A. chasmanthum* for HR-LCMS/MS, and most of the identified compounds are reported for the first time in these species.

DPPH, FRAP, SOR and OH tests were used to analyze the scavenging capacity of radicals in *A. chasmanthum* wild rhizomes. As a result of our observations, the extracts’ high phenolic content was likewise linked to their high levels of radical scavenging. According to our results, methanolic extracts show the highest radical scavenging effect than ethyl acetate and hexane plant extracts. The extracts of wild rhizomes showed radical scavenging activity that was less than all standards at all concentrations, but methanolic extracts showed scavenging activity almost comparable to standards (BHT and OH^−^). Our findings are comparable to those of earlier research [68,69,70]; in radical scavenging tests, the antioxidant effect of several plant extracts was investigated. The phenolic compounds in plant extracts may be responsible for the effect. Bonding/coordination of these molecules with free radicals in the solution may stabilize the DPPH radical or any other radical in the solution. Membrane lipids may be protected against oxidation (lipid peroxidation) caused by peroxides generated in cells by such substances [71]. Furthermore, oxidative stress may contribute to a variety of disorders, including diabetes. *A. chasmanthum* may be able to help with difficulties that occur as a result of an overabundance of reactive oxygen species.

Chemical fungicides are frequently employed to manage fungal diseases, although this practice has been linked to detrimental environmental effects, possible human pesticide exposure and residue deposition on the fruits. However, the emergence of disease resistance on a regular basis has limited the efficiency of synthetic fungicides. As a result, there is a high desire for safer, more effective chemotherapeutic drugs [72,73]. The hunt for natural items with innovative applications, notably, in pest management, is now highly active. Antimicrobial plant extracts containing a range of secondary metabolites, such as quinones, alkaloids, flavonoids, terpenoids, tannins, saponins, and glycosides, have sparked interest in plant disease control studies [36]. Various strategies have been used across the world to manage a severe pathogenic fungus; one key strategy is the use of plant extracts [74,75,76]. Antifungal activity was measured using the poisoned food technique in this study. By supplementing plant extracts into PDA growing medium, the antifungal stability of methanolic and ethyl acetate plant extracts of wild rhizomes of *A. chasmanthum* against fungal strains of *A. niger*, *P. notatum* and *A. flavus*, was assessed. The extracts inhibited the growth of mycelia in accordance with the concentration, with varying degrees of fungal suppression (Table 7). The extracts (ethyl acetate and methanolic) of wild rhizomes showed lesser mycelial inhibition of all pathogenic fungi tested than positive control (hexaconazole) at all concentrations.

Similar findings by Anwar et al. [9] wherein they observed maximum antifungal activity against most of the pathogens with ethyl acetate extracts. Ethyl acetate plant extracts of *A. violaceum* showing strong antifungal activity of almost 95% and 86% against *A. flavus* and *A. niger* [68]. Chloroform extracts from *Aconitum laeve* tubers have antifungal action, suppressing mycelia development by 100 percent in *Fusarium oxysporum* and *Rhizoctonia solani* at 600 μg/mL; whereas, 1200 μg/mL was shown to be effective against *Bipolaris maydis* and *Alternaria alternata* [77]. The methanolic extracts of *A. heterophyllum* had higher antifungal activity against *A. niger* and *Alternaria solani* [78].

## 5. Conclusions

The objective of this work was to find out the therapeutic potential of crude and subsequent fractions extracted from the rhizome of *A. chasmanthum*. The ethyl acetate and methanol fraction have the most promising antifungal, as well as prominent antioxidant, potential. The phytochemical composition of methanolic fraction, which includes alkaloids and has the maximum phenolic and flavonoid yield of phytochemicals, is responsible for these actions, and also demonstrates that flavonoids and phenols have a key role in the plant extract’s antioxidant and antifungal properties. Overall, the methanol fraction derived from *A. chasmanthum* showed significant biological potential and may be formed into different concoctions as a potential novel medicinal source.

## Figures and Tables

**Table 1:** Qualitative phytochemical screening of *Aconitum chasmanthum*.

Constituents	Chemical Tests	Rhizome			Stem			Leaves			Flower		
		H	EA	M	H	EA	M	H	EA	M	H	EA	M
Alkaloids	Sodium hydroxide test Wagner’s test	−	+	++	−	+	++	−	+	++	−	+	++
	Lead acetate test Mayer’s test	−	+	++	−	+	++	−	+	++	−	+	++
Flavonoids	Keller–Kiliani test Sodium hydroxide test	+	+	++	+	−	++	−	+	++	+	+	++
	Fehling’s test Lead acetate test	+	+	++	+	−	++	−	+	+	+	+	++
Glycosides	Phenol’s test Keller–Kiliani test	+	+	+	+	+	++	+	+	+	+	+	++
	Frothing/foam test Fehling’s test	−	+	+	+	+	++	+	+	+	+	+	++
Phenols	Salkowski test Phenols test	+	+	++	−	−	−	−	−	−	+	+	++
Saponins	LB test Frothing/foam test	+	+	++	−	−	−	−	−	−	+	+	++
Steroids	Ferric chloride test Liebermann–Burchard test	+	+	++	+	+	+	+	+	+	+	+	++
Tannin	Salkowski test FeCl_3_ test	−	+	+	+	+	+	−	−	−	+	+	+
Terpenoids	Sodium hydroxide test Salkowski test	−	+	+	+	+	++	−	+	+	+	+	++
Terpenes	Salkowski’s test	−	−	−	−	−	−	−	−	−	+	+	+

Note: ‘++’ = Strong presence, ‘+’ moderate presence, and ‘−’ = absent; where H = Hexane extract; EA = Ethyl acetate Extract; M = Methanol extract, respectively.

**Table 2:** Bioactive compounds and their chemical class identified in *A. chasmanthum* ethyl acetate extract of wild rhizome using HR-LCMS.

S. No.	Ret. Time	*m*/*z*	Adduct	Compound Name	Comp. Formula	Category/Class/Subclass	Exact Mass
1	1.215	180.1013	(M^+^H)^+^	2(N)-Methyl-norsalsolinol	C_10_H_13_NO_2_	Alkaloid	179.0941
2	1.332	377.0898	(M^+^HCOO)^−^	3,3′,5-Trihydroxy-4′,7-dimethoxyflavanone	C_17_H_16_O_7_	Flavonoid derivative	332.0896
3	1.76	376.2468	(M^+^H)^+^	Icaceine	C_22_H_33_NO_4_	Diterpene alkaloid	375.2395
4	1.939	470.273	(M^+^H)^+^	Dimethylaminoethylreserpilinate	C_26_H_35_N_3_O_5_	Reserpilinate derivative alkaloid	469.2659
5	2.16	197.0481	(M^+^H)^−^	Syringic acid	C_9_H_10_O_5_	Phenolic compound	198.0554
6	2.261– 2.635	454.2782	(M^+^H)^+^	Delcosine	C_24_H_39_NO_7_	Diterpene alkaloid	453.271
7	2.319	503.1451	(M^−^H)^−^	6-Caffeoylsucrose	C_21_H_28_O_14_	Glycoside	504.1523
8	2.536	353.0873	(M^+^H)^−^	Chlorogenic acid	C_16_H_18_O_9_	phenolic derivative	354.0945
9	3.434	378.2628	(M^+^H)^+^	Karakoline	C_22_H_35_NO_4_	Diterpene alkaloid	377.2554
10	3.619	408.273	(M^+^H)^+^	Cammaconine	C_23_H_37_NO_5_	Diterpene alkaloid	407.2656
11	4.658– 4.986	468.2945	(M^+^H)^+^	Browniine	C_25_H_41_NO_7_	Diterpene alkaloid	467.2871
12	4.886	422.2891	(M^+^H)^+^	Talatizamine	C_24_H_39_NO_5_	Diterpene	421.2819
13	4.988	480.2943	(M^+^H)^+^	Delcorine	C_26_H_41_NO_7_	Diterpene alkaloid	479.2871
14	5.107	659.1804	(M^+^HCOO)^−^	Catechin 3′,5-diglucoside	C_27_H_34_ O	Flavonoid O-glycosides	614.18
15	5.13–6.038	510.3051	(M^+^H)^+^	Germine	C_27_H_43_NO_8_	Alkaloid	509.2977
16	5.189	355.1577	(M^−^H)^−^	Gingerenone A	C_21_H_24_ O_5_	Polyphenol	355.1577
17	5.27	491.1621	(M^+^HCOO)^−^	Osmanthuside A	C_23_H_26_O_9_	Coumaric acid esters	446.1638
18	5.407– 7.383	494.3118	(M^+^H)^+^	Zygadenine	C_27_H_43_NO_7_	Alkaloid	493.3043
19	5.805	693.2109	(M^−^H)^−^	Sucrose 1′,4′-(4,4′-dihydroxy-3,3′-20dimethoxy-b-truxinate)	C_32_H_38_O_17_	Stilbene glycoside	694.2182
20	5.815	579.1804	(M^−^H)^−^	(S)-Naringenin 8-C-(2″-rhamnosylglucoside)	C_32_H_38_ O_17_	Flavonoid glycoside	580.1874
21	5.97	545.1942	(M^+^CH_3_COO)^−^	Haplodimerine	C_28_H_26_N_2_O_6_	Quinoline alkaloid	486.1804
22	6.032	521.2094	(M^−^H)^−^	Isolariciresinol 9′-O-β-D-glucoside	C_26_H_34_O_11_	Lignan glycoside	522.2163
23	6.352	555.1701	(M^−^H)^−^	7-Dehydrologanin tetraacetate	C_25_H_32_O_14_	Terpene	556.1776
24	7.077	521.1375	(M^−^H)^−^	Sudachiin A	C_24_H_26_O_13_	Flavonoid glycoside	522.1445
25	7.451	616.3103	(M^+^H)^+^	Hypaconitine	C_33_H_45_NO_10_	Diterpene alkaloid	615.3031
26	7.807	533.2579	(M^−^H)^−^	7,8-Dihydrovomifoliol 9-[rhamnosyl-(1->6)-glucoside]	C_25_H_42_O_12_	Flavonoid glycoside	534.2651
27	8.13	630.3276	(M^+^H)^+^	Indaconitine	C_34_H_47_NO_10_	Diterpene alkaloid	629.32
28	8.521– 11.332	630.3276	(M^+^H)^+^	Falaconitine	C_34_H_47_NO_10_	Diterpene alkaloid	629.32
29	9.897– 11.561	630.3276	(M^+^H)^+^	Finaconitine	C_33_H_46_N_2_O_10_	Diterpene alkaloid	629.32
30	12.749	535.1612	(M^+^HCOO)^−^	Edulisin I	C_28_H_26_O_8_	Furanocoumarins	490.1603
31	13.263– 14.128	639.3123	(M^−^H)^−^	N1,N5,N10-Tris-trans-p-coumaroylspermine	C_37_H_44_N_4_O_6_	Coumaric acid derivative	640.3186
32	13.913	563.4011	(M^+^HCOO)^−^	Ganoderiol C	C_32_H_54_O_5_	Triterpenoid	518.4029
33	16.648	593.2809	(M^+^CH3COO)^−^	3-Hydroxy-beta-ionol 3-[glucosyl-(1->6)-glucoside]	C_25_H_42_O_12_	Terpene glycoside	534.2668
34	16.764	473.3327	(M^−^H)^−^	(3-β,17-α,23S)-17,23-Epoxy-3,28,29-trihydroxy-27-norlanost-8-en-24-one	C_29_H_46_O_5_	Triterpenoid	474.3399
35	19.711	597.4014	(M^−^H)^−^	Idoxanthin	C_40_H_54_O_4_	Carotenoid (xanthophyll)	598.4063

**Table 3:** DPPH free radical scavenging activity of different extracts of *A. chasmanthum* rhizome.

Conc. (μg/mL)	Hexane	Ethyl Acetate	Methanol	α-tocopherol
50	12.92 ± 0.73	22.38 ± 0.42	27.99 ± 0.55	50.24 ± 1.10
100	23.42 ± 0.52	31.48 ± 0.54	39.69 ±0.44	55.67 ± 0.87
150	29.46 ± 0.54	38.28 ± 1.05	45.50 ± 0.47	61.24 ± 0.27
200	37.67 ± 0.75	50.23 ±1.42	57.48 ± 0.63	75.37 ± 0.32
250	49.99 ± 1.10	59.59 ± 0.77	65.21 ± 0.95	81.10 ± 1.16
IC_50_ Value	263.01 ± 1.70 μg/mL	199.5 ± 1.99 μg/mL	163.71 ± 2.69 μg/mL	118.79 ± 1.27 μg/mL

Data reported as mean ± SE of three replicates.

**Table 4:** Ferric-reducing antioxidant power (FRAP) values for *A. chasmanthum* rhizomes.

Conc. (μg/mL)	Hexane Extract	Ethyl Acetate	Methanol	Ascorbic Acid
50	7.72 ± 0.41	11.67 ± 0.88	16.31 ± 0.24	32.56 ± 0.10
100	16.68 ± 0.37	25.50 ± 0.96	31.41 ± 0.11	43.55 ± 0.43
150	28.65 ± 1.55	35.33 ± 0.13	42.67 ± 0.14	59.34 ± 0.96
200	41.43 ± 0.15	46.36 ± 0.62	55.54 ± 0.15	70.46 ± 0.51
250	50.26 ± 0.48	54.30 ± 0.75	68.64 ± 0.37	80.25 ± 1.27
IC_50_ value	246.37 ± 0.56 μg/mL	219.71 ± 0.32 μg/mL	179.11 ± 0.73 μg/mL	125.80 ± 0.63 μg/mL

Data reported as mean ± SE of three replicates.

**Table 5:** Superoxide radical scavenging activity of different extracts of *A. chasmanthum* rhizome.

Conc. (μg/mL)	Hexane	Ethyl Acetate	Methanol	BHT
50	12.59 ± 0.38	22.18 ± 0.46	26.79 ± 0.91	34.44 ± 1.31
100	22.83 ± 0.61	30.49 ± 0.89	36.11 ± 1.03	42.35 ± 0.32
150	27.96 ± 0.28	41.54 ± 0.41	46.54 ± 1.01	52.9 ± 0.92
200	39.13 ± 0.34	50.18 ± 1.03	52.46 ± 0.54	60.61 ± 0.53
250	50.86 ± 0.75	54.75 ± 1.07	60.0 ± 0.21	68.39 ± 1.72
IC_50_ Value	243.54 ± 0.34 μg/mL	192.23 ± 0.39 μg/mL	178.33 ± 0.91 μg/mL	122.61 ± 0.57 μg/mL

Data reported as mean ± SE of three replicates.

**Table 6:** Hydroxyl radical scavenging (OH^−^) activity of different extracts of *A. chasmanthum* rhizome.

Conc. (μg/mL)	Hexane	Ethyl Acetate	Methanol	OH^−^
50	6.32 ± 0.29	16.61 ± 0.70	19.21 ± 0.68	37.62 ± 0.39
100	14.01 ± 0.74	28.92 ± 0.40	34.01 ± 0.29	48.81 ± 0.85
150	23.48 ± 1.81	34.01 ± 0.29	48.01 ± 0.59	57.62 ± 0.58
200	35.02 ± 0.88	47.79 ± 1.41	57.17 ± 0.29	68.02 ± 0.49
250	51.86 ± 1.95	58.98 ± 0.70	67.24 ± 0.49	74.68 ± 0.59
IC_50_ value	238.85 ± 0.23 μg/mL	208.85 ± 0.45 μg/mL	159.64 ± 0.42 μg/mL	101.99 ± 0.66 μg/mL

Data reported as mean ± SE of three replicates.

**Table 7:** Antifungal activity (percentage mycelial inhibition) of rhizome extracts of *A. chasmanthum*.

Pathogenic Fungi	% Mean Mycelial Inhibition			Negative Control	Hexaconazole	MIC (μg/mL)
	Concentrations (%) of Culture Filtrate					
	5%	10%	15%			
*Aspergillus niger ME*	41.16 ± 3.32 ^a^	52.94 ± 1.46 ^a^	66.18 ± 1.03 ^b^	25.23 ± 1.13 ^a^	83.76 ± 0.26 ^a^	230
*Aspergillus flavus ME*	53.81 ± 1.11 ^b^	69.36 ± 1.05 ^b^	78.91 ± 1.19 ^c^	15.47 ± 2.23 ^b^	90.53 ± 1.32 ^b^	200
*Penicillium notatum ME*	58.81 ± 0.76 ^b^	71.46 ± 1.06 ^b^	83.14 ± 0.97 ^bc^	12.35 ± 0.73 ^b^	95.65 ± 2.23 ^b^	190
*Aspergillus niger EAE*	49.42 ± 0.99 ^b^	49.98 ± 3.78 ^a^	59.56 ± 3.14 ^a^	25.23 ± 1.13 ^a^	83.76 ± 0.26 ^a^	250
*Aspergillus flavus EAE*	36.75 ± 3.72 ^a^	65.88 ± 1.74 ^b^	74.33 ± 0.71 ^bc^	15.47 ± 2.23 ^b^	90.53 ± 1.32 ^b^	210
*Penicillium notatum EAE*	55.34 ± 1.06 ^b^	69.75 ± 0.99 ^b^	78.21 ± 1.31 ^bc^	12.35 ± 0.73 ^b^	95.65 ± 2.23 ^b^	200

The data were determined for up to 7 days. The data represents the mean value ± SE (standard error) and mean ± SE with followed by different letters within each column were judged to be statistically significant and operational using the Tukey’s test at *p* ≤ 0.05. Tukey’s test shows that the mean ± SE followed by the different letters within each column are substantially different at *p* ≤ 0.05. (ME: methanolic extract; EAE: ethyl acetate extract).

## Data Availability

All data are available in this manuscript.
